# Single Nucleotide Polymorphism (SNP) in the Adiponectin Gene and Cardiovascular Disease

**DOI:** 10.7508/ibj.2016.04.001

**Published:** 2016

**Authors:** Salvatore Chirumbolo

**Affiliations:** Department of Medicine-Unit of Geriatry, LURM est Policlinico GB Rossi, Piazzale AL Scuro 10, 37134 Verona, Italy

Dear Editor,

The recent article by Mohammadzadeh *et al*.[1] on the latest issue of this Journal showed that the T allele +276G/T SNP of *ADIPOQ* gene is more associated with the increasing risk of coronary artery disease (CAD) in subjects with type 2 diabetes. Adipocytes were described in myocardial tissue of CAD patients and their role recently discussed[2,3]. Susceptibility to CAD by polymorphism in the *Q* gene of adiponectin has been reported for 3’-UTR, which harbours some genetic loci associated with metabolic risks and atherosclerosis[4]. Actually, previous studies have shown that the haplotype SNP +276G>T was associated with a decreased risk of CAD, after adjustment for potential confounding factors, therefore some controversial opinion still exists[5]. This evidence should be associated with the role exerted by adipocytes and adiponectin in heart physiology. In particular, in hypertensive disorder complicating pregnancy (HDCP), by investigating the population frequency of alleles, genotypes, and haplotypes of two single nucleotide polymorphisms (SNPs), namely +45T>G (rs2241766) and +276G>T (rs1501299), some authors found that the SNP +276 TT genotype was significantly associated with protection against HDCP, when compared to the pooled G genotypes[6]. Moreover, the same +276G/T SNP haplotype was strongly associated with biliary atresia, an intractable neonatal inflammatory and obliterative cholangiopathy, leading to progressive fibrosis and cirrhosis[7]. CAD is closely related to adiponectin biology. The same isoforms of adiponectin seem to be not associated to CAD severity but to glucose metabolism and its impairment[8]. In the paper by Mohammadzadeh *et* *al*.[1], T allele in +276G/T SNP haplotype is highly associated with CAD in subjects with type 2 diabetes, but this linkage should be reappraised if related much more to diabetes rather than CAD. Association of T allele in the indicated SNP with CAD may be an indirect consequence of type 2 diabetes, as reported by others[9] or a direct marker for CAD affected patients[10]. The paper by Mohammadzadeh *et al*.[1] assesses data coming elsewhere from literature but raises important concerns about the suitability of *ADIPOQ* SNPs in diagnosing susceptibility to CAD and the relationship with plasma adiponectin level. In normal, non diabetic, normoglycemic subject, this relationship does not seem to work. Therefore the question is how much predictive this SNP haplotype may be to foresee metabolic syndrome and CAD onset risk in young health subjects? Maybe, the role of adiponectin in cardiovascular physiology depends on its ability to target adiponectin receptors and to negatively regulate obesity. Some authors reported in healthy volunteers an absence of correlation between circulating adiponectin levels and biochemical markers, particularly lipoproteins and suggested that SNP +276G>T was related to an independent effect on adiponectin levels and on lipoprotein metabolism[11]. On the contrary, adiponectin genetic variants and SNP +276G>T was associated with increasing susceptibility of type 2 diabetes and plasma glucose impairment[12].

The interesting study by Mohammadzadeh *et al*.[1] suggests that SNP of *ADIPOQ* +276G>T should be related to susceptibility to glucose metabolism, while indirectly to lipid metabolism and fat-related cardiovascular damage.
